# Supplementary material for: Identification of photoperiod-induced specific miRNAs in the adrenal glands of Sunite sheep (Ovis aries)
Source: Front Vet Sci. 2022 Jul 22;9:888207. doi: 10.3389/fvets.2022.888207 (PMC9354845; doi:10.3389/fvets.2022.888207)
Supplement: Supplementary file 1 [file Data_Sheet_1.ZIP › Supplementary file/miRDeep2 file.zip!/miRDeep2 file/Novel_Structure/SP42A3/Novel_113.pdf]

Provisional ID	: NC_019460.2_8944
Score total	: 53.8
Score for star read(s)	: 3.9
Score for read counts	: 46.5
Score for mfe	: 1.8
Score for randfold	: 1.6
Score for cons. seed	:
Total read count	: 103
Mature read count	: 102
Loop read count	: 0
Star read count	: 1

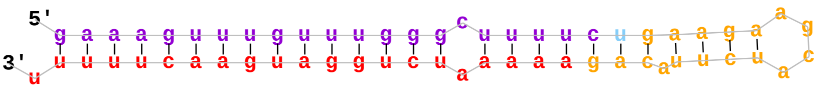

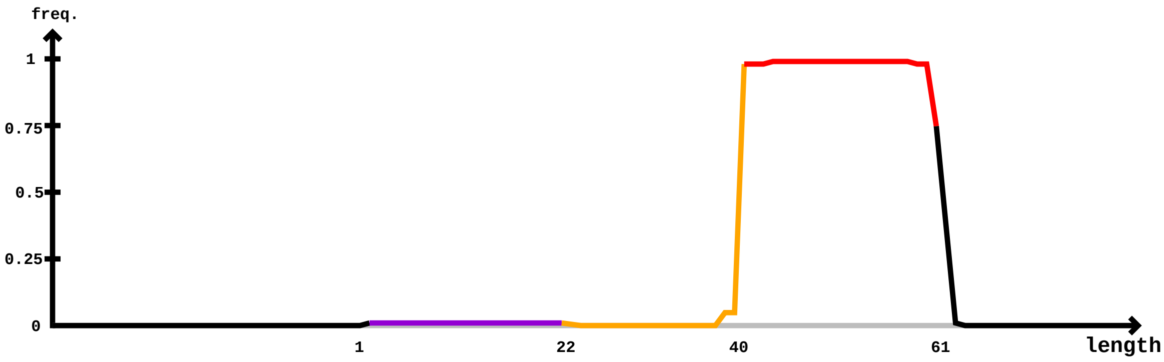

Star Mature

```
5'- cugaaaaacuuugauuauacugaguuggccagaaaguuuguuugggcuuuucugaagaagcaucuuacagaaaaaucuggaugaacuuuuuggccaauccaacaauauauu   -3'    obs
    cugaaaaacuuugauuauacugaguuggccagaaaguuuguuugggcuuuucugaagaagcaucuuacagaaaaaucuggaugaacuuuuuggccaauccaacaauauauu          exp
    ....................((.(((((((((((((((((((((((.(((((((((((....)))).))))))).))))))))))))))))))))))).))..........    reads    mm    sample
    ...............................gaaaguuuguuugggUuuuuc...........................................................    1        1     seq
    ....................................................................agaaaaaucugAaugaacuuuuu....................    5        1     seq
    ......................................................................aaaaaucugAaugaacuu.......................    1        1     seq
    ......................................................................aaaaaucugAaugaacuuuu.....................    24       1     seq
    ......................................................................aaaaaucugAaugaacuuuuu....................    69       1     seq
    ......................................................................aaaaaucuggaugaacuuuuu....................    2        0     seq
    .........................................................................aaCcuggaugaacuuuuugg..................    1        1     seq
```
